# Defining and measuring unmet palliative care needs among people with life-limiting illness: A scoping review of international evidence

**DOI:** 10.1177/02692163261416279

**Published:** 2026-02-06

**Authors:** Therese Johansson, Melanie F. J. Diggle, Anne Finucane, Joanna M. Davies, Irene J. Higginson, Katherine E. Sleeman, Lorna K. Fraser, Fliss E. M. Murtagh, Anna E. Bone

**Affiliations:** 1 Cicely Saunders Institute of Palliative Care, Policy & Rehabilitation, Florence Nightingale Faculty of Nursing, Midwifery & Palliative Care, King’s College London, London, UK; 2 Clinical Psychology, School of Health in Social Science, University of Edinburgh, Edinburgh, UK; 3 Wolfson Palliative Care Research Centre, Hull York Medical School, University of Hull, Hull, UK

**Keywords:** needs assessment, health services needs and demand, terminal care, hospice care, scoping review

## Abstract

**Background:**

Quantifying palliative care needs and whether they are met is essential for effective service planning and provision. Estimates of palliative care needs are widely reported but less is known about *unmet* needs, with no accepted definition of this construct or guidance on how to measure it.

**Aim:**

To investigate how unmet palliative care needs for adults with advanced life-limiting illness have been defined, measured, and reported in the evidence.

**Design:**

Scoping review following the Joanna Briggs Institute guidelines: protocol registered on Open Science Framework (10.17605/OSF.IO/M8DHA).

**Data sources:**

In October 2024, we searched MEDLINE, EMBASE, CINAHL, and PsycINFO for quantitative and mixed-methods studies published after 2000, with backward and forward citation and manual searching of grey literature. Data on definitions, measurement and reporting of unmet needs were extracted, charted, and summarised narratively using inductive content analysis and framework synthesis.

**Results:**

Seventy studies were included: 9 reviews and 61 primary evidence studies from 16 countries. Only 11 (16%) studies explicitly defined unmet palliative care needs. We identified three approaches to measurement of unmet palliative care needs: (1) prevalence of *symptoms and concerns*; (2) *access to services* (e.g. specialist palliative care); and (3) *sufficiency of service provision to resolve symptoms and concerns*.

**Conclusions:**

This novel review identifies a lack of consensus in defining, measuring and reporting unmet palliative care needs. We propose three distinct approaches to conceptualising unmet palliative care needs and outline their strengths and limitations. Practical guidance on their use is provided to support researchers, clinicians, and policymakers in selecting appropriate approaches for assessment and reporting.

**What is already known about the topic?**

- Existing research and policy often report prevalence of palliative care needs with limited consideration to the extent to which needs are met or unmet.
- Assessing and quantifying *unmet* palliative care needs is critical for guiding healthcare planning and delivery, but clear guidance on how this construct should be defined and measured is lacking.

**What this paper adds**

- Few studies provide a clear, detailed definition of unmet palliative care needs.
- We identify three main approaches to measuring unmet needs in palliative care research, by quantifying: (1) Symptoms and concerns; (2) Access to services; and (3) Sufficiency of service provision to resolve symptoms and concerns.
- There is little focus on non-cancer populations and few reports of involvement of patients and carers in studies measuring unmet palliative care needs.

**Implications for practice, theory, or policy**

- Methodological strengths and limitations of the three identified approaches to measuring unmet palliative care needs are discussed.
- To address the knowledge gaps identified, recommendations for reporting of definitions and how these are operationalised are provided.

## Background

With population ageing, rising multimorbidity, and increasing health-related suffering,^1–4^ providing timely and adequate palliative care is becoming a major public health priority. Despite palliative care being recognised as a human right,^5^ barriers to access persist, leaving some needs unmet. Assessing and quantifying unmet palliative care needs of a population is essential to inform effective planning and delivery of services and reduce inequitable access,^6–8^ yet guidance on how best to define and measure this construct is lacking.

Unmet healthcare needs are a concern at individual, group, and societal levels, as they are associated with poorer quality of life, adverse health outcomes, increased risk of hospitalisation and urgent care use, and higher healthcare costs.^9–11^ In healthcare research, care needs are commonly defined using Bradshaw’s typology as the “ability to benefit from healthcare services.”^12^ Care needs can be further categorised as: (1) *felt need* (individual perceptions of need); (2) *expressed need* (perceived need resulting in demanded care); (3) *normative need* (defined by experts and thus largely based on healthcare professionals’ perceptions), and (4) *comparative need* (needs compared across different groups).^12^ While other frameworks for conceptualising need exist, for example, Maslow’s hierarchy of need, Bradshaw’s typology is particularly useful in palliative care as it highlights who determines the need (which influences what is reported as need) rather than simply the what the need is.^13^

A needs assessment is a process to determine need for care services in relation to health problems, either at the individual level (e.g. clinical assessments of patients’ healthcare needs) or population-level (e.g. evaluating community needs for care services).^14^ A review by Franks et al.^15^ identified two main methods for population-level palliative care needs assessment. The first is an epidemiological approach that estimates needs in a patient population based on mortality data of specific life-limiting conditions that may require palliative care and symptom prevalence among patients in advanced stages of these illnesses. The second is an approach based on healthcare service use, which assesses palliative care needs by analysing patterns in service usage data, such as hospice admissions or referrals to home care teams. However, both approaches have limitations. The epidemiological approach relies on mortality and symptom data that might not be readily available and does not specify care required to meet palliative care needs. The service use approach, on the other hand, only captures patients already accessing care, thereby overlooking those not known to palliative services.

It is also vital to understand how many do not have their palliative care needs met. Reported prevalence of unmet palliative care needs in the literature is inconsistent, largely due to methodological variability. A systematic review by Harrison et al.^16^ showed that between 1% and 93% of people with cancer have unmet palliative care needs. Similarly wide prevalence estimates across care domains have been reported by Wang et al.^17^ The lack of an accepted definition of unmet palliative care needs makes estimations difficult, especially at the population-level.

The present review expands on Franks et al.’s findings by focussing on *unmet* palliative care needs while broadening the scope beyond prior reviews, which have often focussed on unmet needs in specific patient groups or care settings.^18,19^ To inform our search strategies for, we constructed operational definitions of “palliative care needs” and “unmet palliative care needs” from definitions of needs and unmet needs in the broader health and care literature^20–23^ and the World Health Organisation’s classification of palliative care as a holistic care approach to improve the quality of life of patients with life-limiting illness and their families.^5,24^ To operationalise this, we defined palliative care needs as “the ability for people with life-limiting illness to benefit from available care to manage a range of symptoms and concerns including physical, psychological, social, spiritual, informational, and practical.” These needs can be addressed by specialist and/or generalist palliative care professionals, or a combination of both.^25^ We defined unmet palliative care needs as “the difference or gap between required or expected care and actual care received by people with life-limiting illness.”

This review aimed to clarify concepts in the literature by addressing the question: How have unmet palliative care needs for adults with advanced life-limiting illness been defined, measured, and reported in the evidence?

## Methods

### Design

This scoping review follows the methodological guidelines set out by the Joanna Briggs Institute (JBI)^26^ and is reported according to the Preferred Reporting Items for Systematic Reviews and Meta Analyses extension for Scoping Reviews (PRISMA ScR).^27^ The review protocol was registered on the Open Science Framework (https://doi.org/10.17605/OSF.IO/M8DHA).

### Eligibility criteria

We included studies reporting quantitative data on how unmet palliative care needs of adults (aged 18+ years) with advanced life-limiting illness were defined, measured and reported. Table 1 presents the full inclusion and exclusion criteria. Peer-reviewed quantitative and mixed-methods studies were included, as were systematic and scoping reviews reporting results from quantitative and mixed-methods studies. Grey literature (reports but not published abstracts) that quantitatively measured unmet palliative care needs was also included. Only English-language sources were included. To ensure that the review built on the review by Franks et al.,^15^ we only included papers and grey literature evidence published in 2001 or after.

**Table 1.** Inclusion and exclusion criteria for the literature database searches.

Study characteristic	Inclusion criteria	Exclusion criteria
Type of participants	Adults, over 18 with: - Advanced illness - Life-limiting illness - End-stage illness For example advanced cancer (stages 3 or 4), end-stage renal disease (stage 5), heart failure (class 3 or 4), advanced dementia (stage 6 or 7) Carers reporting on behalf of adult patients (e.g. bereaved family carers) were included if the remaining inclusion criteria were met.	Adults, over 18 who are: - In early disease stages - Survivors - In remission - Not otherwise specified as having advanced illness Children, under 18 Carers reporting on their own unmet palliative care needs Carers reporting on behalf of children or adolescents
Concept	Unmet palliative/supportive/end-of-life care needs, including measures of single care domains of unmet palliative care needs (e.g. unmanaged pain or unmet spiritual care needs).	Unmet bereavement support needs only
		Family carers’ unmet support needs only
		Study focus is solely on psychometric properties of measurements
Context	Any geographic location, setting, or health system.	No study was excluded based on context.
Type of evidence	Quantitative or mixed-methods original research studies of any design reporting quantitative data	Qualitative original research studies
	Quantitative or mixed-methods systematic or scoping reviews that report quantitative data	Qualitative systematic or scoping reviews
		Opinion pieces
		Conference abstracts
		Published protocols

### Search strategy

An initial PubMed scoping search of identified relevant published articles and identify target papers. Keywords from titles and abstracts and index terms assigned to the articles from the initial search informed the final search strategy for MEDLINE (Ovid), EMBASE (Ovid), CINAHL (EBSCO) and PsycINFO (Ovid; see Supplemental File 1). The search strategy was developed in collaboration with an information specialist and adapted for each database and/or information source. Target papers for inclusion in the search strategy were set to check the robustness of the search. All databases were searched on 2 October 2024.

Grey literature was hand-searched by one reviewer (MD) across relevant websites (Hospice UK, National Council for Palliative Care, Marie Curie, Macmillan Cancer Support, The Kings Fund, The Nuffield Trust, RAND and RAND Europe, The Lien Foundation, The Health Foundation, The Strategy Unit and World Health Organisation). Additional grey literature was identified through Open Grey and Google Scholar (using the first 100 references).^26^ Forward citation searching of included studies was undertaken using Scopus.

### Study selection

All papers identified from the searches were imported into Covidence and duplicates removed. Titles and abstracts were screened against the inclusion criteria by a single reviewer (TJ/MD/LF/AF), with a subset of 20% papers independently screened by a second reviewer to ensure screening consistency. Full-text screening was conducted by a single reviewer (TJ/MD), with 20% independently screened by a second reviewer (AF/LF) to check agreement. Any disagreements were resolved through discussion with a third reviewer.

### Data extraction and charting

Data from eligible papers and evidence sources were extracted verbatim, where possible, by a single reviewer (TJ/MD/AB) using a bespoke data extraction form in Covidence (see Supplemental File 2). A second reviewer (AB/AF) independently checked 20% of the extracted data for accuracy. Any discrepancies between the reviewers were settled through discussion with a third reviewer.

Our extraction form was adapted from the JBI template^26^ to fit the review aim and capture data on study characteristics (e.g. authors, publication year, study design, population) and how unmet palliative care needs were defined, measured, and reported (e.g. presentation of study findings of prevalence of unmet needs). For included systematic reviews, only review authors’ definitions and operationalisations of unmet palliative care needs, and conclusions regarding its measurement were extracted (excluding primary study results). Data were extracted at study-level; if several papers reported the same study, only one set of data was extracted. Data were recorded and analysed in Microsoft Excel.

### Data analysis

Following scoping review guidance, no quality appraisal was conducted, as the aim was to provide a descriptive overview of the available evidence rather than assess study quality.^28,29^ Data analysis focussed on summarising relevant study findings, both by charting and by narrative summary using inductive content analysis.^26,30^ Whether studies defined unmet palliative care needs or not was inductively coded as “yes” (a clear explanation of the construct within a palliative or supportive care context), “partially” (addressing unmet needs within a palliative or supportive care context, often using prior research findings suggesting issues with palliative care access, but without explicitly delineating the construct) and “no” (no explanation of the construct).

To identify and map the dimensions of unmet palliative care needs captured in existing measures, we conducted a framework synthesis^26,31^ based on the domains in Goni-Fuste et al.’s^32^ review of comprehensive palliative care needs assessments, encompassing *Physical*, *Psychological*, *Spiritual*, *Social*, *Information*, *Financial*/*legal* (e.g. preparing wills), *Practical* (e.g. household tasks), *Autonomy* (e.g. dependency on others), *Role activities* (e.g. difficulties with employment), *Personal issues* (e.g. handling personal affairs), *Healthcare* (e.g. support from healthcare professionals). Domains were added as needed.^31^

## Results

**Figure 1.**
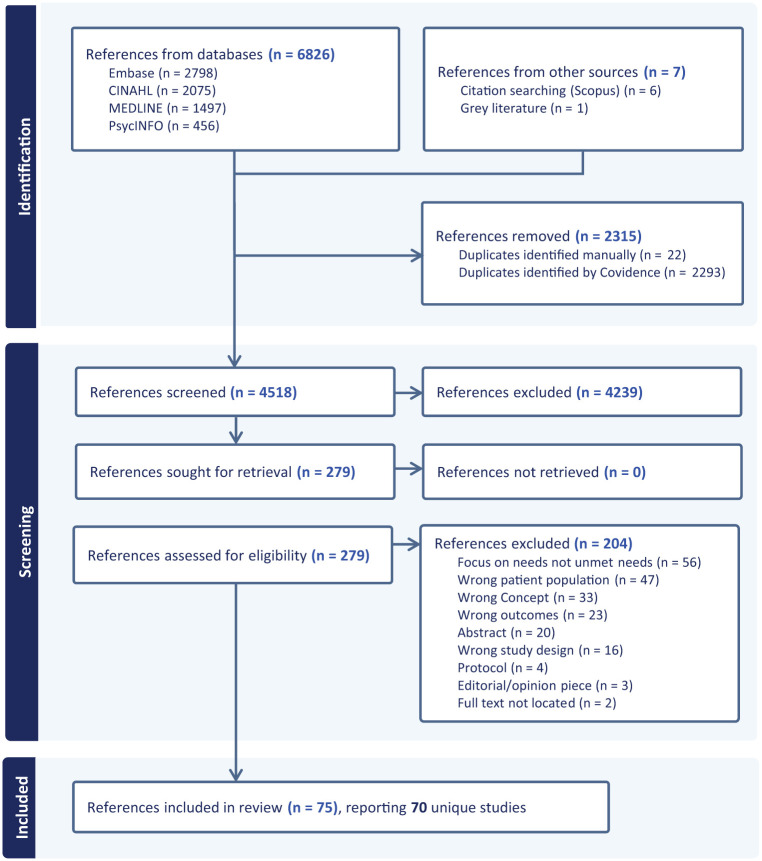
PRISMA flow diagram of study selection.

The searches and study inclusion are presented in a PRISMA flow diagram in Figure 1.^33^ The database searches identified a total of 4513 references after duplicates were removed. Following the title and abstract screening, 274 references were sought for full-text review. After full-text screening against inclusion and exclusion criteria, 75 references were included, reporting on 70 unique studies.

The 70 included studies were published between 2001 and 2024. In total, 61 were primary evidence studies (i.e. original research),^34–95^ and nine were reviews.^16,17,96–102^ The original studies (*n* = 61) were conducted in 16 countries, primarily Western countries such as the United States (*n* = 23), Australia (*n* = 9), Canada (*n* = 5), and the United Kingdom (*n* = 5).

## Study descriptives

### Study designs

Details of study characteristics (*n* = 70) are presented in Table 2. Most included studies used observational designs: commonly cross-sectional (*n* = 43, 59%), prospective cohort (*n* = 8, 11%), and retrospective cohort (*n* = 6, 9%) designs. The primary evidence studies that directly measured unmet palliative care needs, mostly used convenience samples (*n* = 49, 80%). Only two primary evidence studies reported on patient and public involvement (PPI): one mentioned PPI input when developing the measure,^91^ and one other pilot-tested their questionnaire to ensure it was not too distressing.^36^

**Table 2.** Characteristics of included studies (n = 70), ordered by study design.

Authors and year	Country	Summary of study aim	Study design	Population	Study setting	Sample	Recruitment	Sample size
Prospective cohort studies								
Faris et al.^a^ (2024)	Australia	Assess unmet needs among patients with brain tumours and explore feasibility of addressing these in clinical practice	Prospective cohort study	Advanced cancer	Hospital	Convenience sample	Clinic patients	116
Hamano et al. (2023)	Japan	Assess unmet needs among non-cancer patients across the illness trajectory and factors related to these	Prospective cohort study (multi-centre)	Unspecified life-limiting illness	Care home	Convenience sample	Clinic patients	785 (T0); 553 (T1); 370 (T2); 317 (T3)
Hampton et al. (2007)	United States	Assess spiritual needs among advanced cancer patients and whether they were met	Prospective cohort study	Advanced cancer	Hospice	Convenience sample	Clinic patients	90
Kiely et al. (2010)	United States	Assess how bereaved family member’s experiences of hospice care is associated with symptom treatment and unmet care needs in the last days of life	Prospective cohort study	Dementia	Care home	Convenience sample	Clinic patients	323
Kirkland et al.^b^ (2021)	Canada	Compare characteristics between patients who have unmet palliative care needs and those who do not	Prospective cohort study	Unspecified life-limiting illness	Hospital	Convenience sample	Clinic patients	663
Makaroun et al. (2018)	United States	Examine how late healthcare transitions are associated with unmet needs, communication and quality of care	Prospective cohort study	Decedents (any site of death and cause of death)	Any care setting	Nationally representative sample	National Health and Aging Trends Study register	1653
Sharpe et al. (2005)	Australia	Assess unmet needs in a patient group and explore if ratings differ between patients, their families, and healthcare professionals	Prospective cohort study	Advanced cancer	Hospital	Convenience sample	Clinic patients	142
Vogt et al. (2021)	Germany	Assess symptom burden and palliative care needs during the first year of diagnosis with incurable cancer	Prospective cohort study	Advanced cancer	Hospital (cancer centre)	Convenience sample	Clinic patients	161
Retrospective cohort studies								
Arabadzhyan et al. (2024)	United Kingdom	Explore unmet care need in the final months of life among people who died of cancer during the Covid-19 pandemic	Retrospective cohort study	Advanced cancer	Any care setting	Population-based sample	National mortality register	130,490
Axelsson et al. (2018)	Sweden	Assess symptom prevalence, relief, and management among patients with end-stage kidney disease	Retrospective cohort study	End-stage renal disease	Any care setting	Population-based sample	National palliative register	472
Hildenbrand et al. (2022)	United States	Explore the association between cancer patients’ distress and urgent healthcare use	Retrospective cohort study	Advanced cancer	Hospital (cancer centre)	Convenience sample	Clinic patients	848
Hwang et al. (2019)	United States	Characterise the outcomes for patients with end-stage renal disease, define patterns of palliative care use and identify unmet needs	Retrospective cohort study	End-stage renal disease	Hospital	Convenience sample	Medical records from patient register	9363
Safabakhsh et al. (2023)	United States	Calculate unmet need for palliative care among hospitalised patients	Retrospective cohort study	Unspecified life-limiting illness	Hospital	Population-based sample	Clinic patients	92,047
Westley-Wise et al. (2022)	Australia	Assess the unmet need for palliative and end-of-life care, and explore sociodemographic and diagnostic factors associated with suboptimal access to these services	Retrospective cohort study	Decedents (any site of death and cause of death)	Hospital: Community health centre	Population-based sample	Medical records from patient register	3175
Experimental study								
Paterson et al. (2018)	United Kingdom	Explore the effect of an intervention on unmet needs among advanced prostate cancer patients	Randomised controlled trial	Advanced cancer	Hospital	Random sample	Clinic patients	47
Mixed-methods studies								
Paterson et al.(2017)	United Kingdom	Assess unmet care needs among advanced prostate cancer patients	Mixed-methods study	Advanced cancer	Hospital (cancer centre)	Convenience sample	Clinic patients	31
Spence et al. (2020)	Canada	Describe the symptom burden of patients during consultation with an integrated palliative radiotherapy service	Mixed-methods study	Advanced cancer	Hospital (cancer centre)	Convenience sample	Clinic patients	607
Teno et al. (2021)	United States	Examine bereaved relatives’ perceptions of care received in the last month of life	Mixed-methods study	Deaths from any non-traumatic causes	Any care setting	Convenience sample	Mail	623
Cross-sectional studies								
Abu-Odah et al. (2022)	Palestine	Explore cancer patients’ needs	Cross sectional study	Advanced cancer	Hospital	Convenience sample	Clinic patients	379
Alnajar et al. (2024)	Jordan	Assess and compare unmet needs between cancer and non-cancer patients	Cross sectional (comparative) study	Advanced cancer; Non-cancer chronic conditions	Hospital	Convenience sample	Clinic patients	458
Anderson et al. (2001)	United Kingdom	Determine the main concerns experienced by patients with terminal illness and if they were met	Cross sectional study	Palliative care patients (unspecified life-limiting illness); Heart failure	Palliative care services (hospital, hospice, & community-based); Hospital (outpatient clinic)	Convenience sample	Clinic patients	279
Aranda et al. (2005)	Australia	Identify patterns of support and information needs among cancer patients	Cross sectional study	Advanced cancer	Hospital	Convenience sample	Clinic patients	105
Blindbaek et al. (2014)	Denmark	Measure prevalence of problems experiences by patients with advanced kidney disease and whether these were met	Cross sectional study	End-stage renal disease	Hospital	Convenience sample	Clinic patients	27
Buzgova et al. (2014)	Czech Republic	Determine associations between unmet needs, quality of life, anxiety and depression among cancer patients	Cross sectional study	Advanced cancer	Hospital	Convenience sample	Clinic patients	93
Buzgova et al.^c^ (2016)	Czech Republic	Identify unmet palliative care needs among hospitalised patients and explore factors related to these	Cross sectional study	Unspecified life-limiting illness	Hospital	Convenience sample	Clinic patients	349
Chuang et al. (2017)	United States	Estimate generalist and specialist palliative care delivery, estimate unmet needs among inpatients and explore ethnic disparities in palliative care delivery	Cross sectional study	Unspecified life-limiting illness	Hospital	Convenience sample	Clinic patients	8301 (sub-sample (*n* = 401)for in-depth analysis)
Connor et al. (2005)	United States	Assess differences in care among hospice providers	Cross sectional study	Hospice decedents (any cause of death)	Hospice	Convenience sample	Mail	29,292
Cooper et al. (2021)	Australia	Determine how many hospital inpatients would benefit from palliative care and how many were receiving it	Cross sectional (prevalence) study	Unspecified life-limiting illness	Hospital	Convenience sample	Clinic patients	270
Cooper et al. (2024)	Australia	Determine how many hospital inpatients would benefit from palliative care and how many were receiving it	Cross sectional (prevalence) study	Unspecified life-limiting illness	Hospital	Convenience sample	Clinic patients	279
Currow et al. (2008)	Australia	Determine and compare perceived unmet palliative care needs across four subpopulations: those with non-cancer diagnoses; those 75 years and older; those with lower household incomes; and those of non-English speaking backgrounds	Cross sectional (comparative) study	Unspecified life-limiting illness	Specialist palliative care service	Nationally representative sample	Post-bereavement study	7105
DeGroot et al. (2023)	United States	Examine palliative care needs among patients with heart failure	Cross sectional study	Heart failure	Hospital	Convenience sample	Clinic patients	286
Driessen et al. (2023)	the Netherlands	Gain insight into disease- and care-related needs among patients with advanced cancer and explore factors related to these	Cross sectional study	Advanced cancer	Hospital	Convenience sample	Clinic patients	1103
Effendy et al. (2015)	Indonesia and the Netherlands	Determine and compare unmet needs between patients with advanced cancer in Indonesia and the Netherlands	Cross sectional study	Advanced cancer	Hospital; Care home	Convenience sample	Clinic patients	274
Hasegawa et al. (2016)	Japan	Assess unmet needs among cancer patients and factors related to these	Cross sectional study	Advanced cancer	Hospital	Convenience sample	Clinic patients	45
Hasegawa et al. (2021)	Japan	Assess unmet rehabilitation needs among hospice inpatients and how this related to medico-psychosocial factors	Cross sectional study	Advanced cancer	Palliative care service	Random sample	Post-bereavement study	416
Huang et al. (2020)	Singapore	Assess care needs, functional status and quality of life among lung cancer patients aged 50 and older	Cross sectional study	Advanced cancer	Hospital (cancer centre)	Convenience sample	Clinic patients	103
Husain et al. (2013)	Canada	Describe continuity of care and care needs among advanced lung cancer patients and factors related to these	Cross sectional study	Advanced cancer	Hospital	Convenience sample	Clinic patients	116
Hwang et al. (2004)	United States	Assess unmet needs among advanced cancer patients and factors related to these	Cross sectional study	Advanced cancer	Hospital	Convenience sample	Clinic patients	296
Jeyasingam et al. (2008)	Australia	Assess unmet ADL needs among inpatients in a palliative care unit and explore congruence ratings by patients, families and care professionals	Cross sectional (prevalence) study	Palliative care inpatients (any life-limiting illness)	Palliative care service	Convenience sample	Clinic patients	30 Patient-caregiver dyads
Johnsen et al. (2013)	Denmark	Assess the adequacy of help from healthcare services to resolve problems experienced by patients with advanced cancer	Cross sectional study	Advanced cancer	Hospital	Nationally representative sample	Medical records from patient register	977
Kavalieratos et al. (2014)	United States	Assess and compare unmet needs between community-dwelling patients with heart failure and cancer	Cross sectional (comparative) study	Advanced cancer; Heart failure	Palliative care service	Convenience sample	Clinic patients	1031
Khan et al. (2012)	Canada	Assess unmet needs among palliative radiotherapy patients and explore if ratings differ between patients and their families	Cross sectional study	Advanced cancer	Hospital (cancer centre)	Convenience sample	Clinic patients	40
Miniotti et al. (2019)	Italy	Assess care needs, quality of life, and psychological morbidity of patients with advanced colorectal cancer	Cross sectional study	Advanced cancer	Hospital	Convenience sample	Clinic patients	203
Munn et al. (2006)	United States	Explore if hospice care for nursing home residents is associated with symptom management, personal care, spiritual support, and care satisfaction.	Cross sectional study	Care home residents (unspecified life-limiting illness)	Care home	Convenience sample	Phone	124
Oh et al. (2019)	South Korea	Assess unmet needs among patients with amyotrophic lateral sclerosis (ALS) and determine its relation to functional status and quality of life	Cross sectional study	ALS	Hospital	Convenience sample	Clinic patients	186
Osse et al. (2005)	the Netherlands	Assess needs among cancer patients and whether these were met	Cross sectional study	Advanced cancer	Any care setting	Convenience sample	Clinic patients	94
Park et al. (2017)	United States	Assess spiritual needs among heart failure patients	Cross sectional study	Heart failure	Hospital (outpatient clinic)	Convenience sample	Clinic patients	111
Pearce et al. (2012)	United States	Assess the prevalence of insufficient spiritual care and its association with patient-reported outcomes	Cross sectional study	Advanced cancer	Hospital	Convenience sample	Clinic patients	150
Rachakonda et al. (2015)	Australia	Assess unmet needs in cancer patients living in rural areas.	Cross sectional study	Advanced cancer	Community (rural care centre)	Convenience sample	Clinic patients	75
Rhodes et al. (2012)	United States	Examine bereaved family members’ satisfaction with hospice care depending on the proportion of African American patients in hospice.	Cross sectional study	Hospice decedents (any cause of death)	Palliative care service (hospice)	Convenience sample	Mail	11,892
Schenker et al. (2014)	United States	Examine the association between unmet needs and interest in seeing a palliative care physician	Cross sectional study	Advanced cancer	Palliative care service (cancer centre with palliative care clinic)	Convenience sample	Clinic patients	169
Strupp et al. (2018)	Germany	Characterise symptoms of people severely affected by Parkinson Disease/Atypical Parkinsonism and describe their prevalence of unmet needs	Cross sectional study	Parkinson’s Disease	Any care setting	Convenience sample	Magazine advertisement	814
Szekendi et al. (2016)	United States	Estimate the need for a palliative care referral among hospital inpatients, determine the proportion of who receives a referral for palliative care consultation, explore organisational characteristics influencing referral	Cross sectional (prevalence) study	Unspecified life-limiting illness	Hospital	Random sample	Clinic patients	2119
Teno et al. (2004)	United States	Evaluate the dying experience at home and in institutional settings	Cross sectional study	People who died from any chronic illness	Any care setting	Nationally representative sample	Post-bereavement study	1578
Teno et al. (2007)	United States	Examine the association of perceived quality of care in hospice with length of stay and timing of referral	Cross sectional study	Hospice decedents (any cause of death)	Palliative care service (hospice)	Convenience sample	Mail	106,514
Teno et al. (2011)	United States	Examine the perceived effectiveness of hospice services for people dying with dementia	Cross sectional study	Dementia	Any care setting	Random sample	Phone	538
Trandel et al. (2019)	United States	Determine the prevalence of existential distress by identifying unmet existential needs and explore associations with symptom burden	Cross sectional study	Cystic fibrosis	Hospital (outpatient clinic)	Convenience sample	Clinic patients	164
Wang et al. (2021)	China	Examine palliative care needs among advanced cancer patients and their informal caregivers	Cross sectional study	Advanced cancer	Hospital	Convenience sample	Clinic patients	428
Wang et al. (2023)	China	Identify palliative care needs of patients with end-stage renal disease undergoing maintenance haemodialysis, and explore the influencing factors of unmet needs	Cross sectional study	End-stage renal disease	Hospital (outpatient clinic)	Convenience sample	Clinic patients	305
Watson et al. (2019)	United Kingdom	Examine experiences of care and care needs among pancreatic cancer patients	Cross sectional study	Advanced cancer	Community (NHS centre)	Convenience sample	Clinic patients	274
Wegier et al. (2021)	Canada	Measure the prevalence of unmet palliative needs among patients estimated to be in their last year of life	Cross sectional study	People estimated to be in their final year of life (any life-limiting illness)	Hospital (quaternary healthcare facility)	Convenience sample	Clinic patients	403
Systematic reviews								
Bore et al. (2024)	Review conducted in Australia	Determine the prevalence of unmet care needs among cancer patients and explore factors contributing to these	Systematic review	Advanced cancer	Any care setting in Sub-Saharan Africa	-	-	-
Chen et al. (2020)	United States	Identify ways to measure unmet needs for symptom management and communication in a palliative population	Systematic review	Unspecified life-limiting illness	Not stated	-	-	-
Fu et al. (2020)	Review conducted in Australia	Assess care and unmet care needs and factors related to these	Systematic review	Advanced cancer	Not stated	-	-	-
Harrison et al. (2009)	Review conducted in Australia	Assess unmet needs among cancer patients across the illness trajectory, explore associated factors and describe study designs used to research this	Systematic review	Advanced cancer	Any care setting	-	-	-
Hart et al. (2022)	Review conducted in Australia	Assess unmet needs among advanced cancer patients	Systematic review	Advanced cancer	Not stated	-	-	-
Moghaddam et al. (2016)	Review conducted in the United Kingdom	Assess unmet needs among advanced cancer patients, identifying domains of care and instruments to measure unmet need.	Systematic review	Advanced cancer	Not stated	-	-	-
Schmidt et al. (2023)	Review conducted in Switzerland	Identify validated tools for clinicians to capture patient reports on unmet needs, to guide palliative care interventions	Systematic review	Unspecified life-limiting illness	Any care setting	-	-	-
Ventura et al. (2014)	Review conducted in Australia	Summarise the literature on unmet needs of palliative home care patients and carers	Systematic review	Unspecified life-limiting illness	Palliative care service (home care)	-	-	-
Wang et al. (2018)	Review conducted in Hong Kong	Identify the unmet care needs and their associated factors in patients with advanced cancer and their informal caregivers, and summarise the tools used in the included studies	Systematic review	Advanced cancer	Any care setting	-	-	-

a Study also reported in Halkett et al. (2015).^103^

b Study also reported in Kirkland et al.^18^ and Kruhlak et al. (2021).^104^

c Study also reported in Buzgova & Sikorova (2015).^105^

### Populations and settings

As shown in Table 2, most studies (*n* = 45, 64%) focussed on unmet palliative care needs within a single disease group, commonly advanced cancer (*n* = 36, 51%), with three (3%) studies comparing two groups. One-third of studies (*n* = 23, 33%) included patients or decedents with any life-limiting illness. Study settings varied: most studies used hospital settings (*n* = 40, 57%; including cancer centres and outpatient clinics), whereas 11 (16%) focussed on palliative care services (including hospices, hospital units, and home care). Eleven (16%) studies included participants from any care setting, and four (6%) did not specify a setting.

### Definitions of unmet palliative care needs

Details of how studies defined, measured, and reported unmet palliative care needs are presented in Table 3. Only 11 (16%) studies explicitly provided a definition of unmet palliative care needs, whereas 23 (33%) partially defined the concept – often with reference to prior research on care needs. More than half (*n* = 36, 51%) provided no definition.

**Table 3.** Study definitions, measurement and reporting of unmet palliative care needs in the included studies (n = 70).

Study reference	Unmet needs defined	Approach to measurement*	Type of need	Sources of information for measurement	Measure	Timing of measurement	Domains of need‡
Prospective cohort studies							
Faris et al.^a^ (2024)	Partially	(3) Sufficiency of service provision to resolve symptoms and concerns	Felt	Patient reports of symptoms or concerns and if these were resolved by care provision	The 9-item SCNS-Screening Tool (SCNS-ST9), a brief version of the 34-item Supportive Care Needs Survey-Short Form (SCNS-SF34)	Past month	Physical; Psychological; Information; Healthcare; Sexuality
Hamano et al. (2023)	Partially	(1) Symptoms and concerns	Felt	Healthcare staff reports of symptoms or concerns	Integrated Palliative Care Outcome Scale (IPOS)	Past month	Physical; Psychological; Information; Practical
Hampton et al. (2007)	No	(3) Sufficiency of service provision to resolve symptoms and concerns	Felt	Patient reports of spiritual needs and whether these were met or not	The Spiritual Needs Inventory (SNI)	No time frame stated for unmet need measurement	Spiritual
Kiely et al. (2010)	No	(3) Sufficiency of service provision to resolve symptoms and concerns	Felt	Family members’ reports of symptoms or concerns; care service use data	A modified version of the Toolkit After-Death Bereaved Family Member Interview	Last week of life	Psychological; Information; Practical
Kirkland et al.^b^ (2021)	No	(2) Access to services	Normative	Healthcare staff assessments based on clinical characteristics; care service use data	Medical records	No time frame stated for unmet need measurement	Physical; Healthcare
Makaroun et al. (2018)	No	(3) Sufficiency of service provision to resolve symptoms and concerns	Felt	Family members’ reports of symptom or concerns	Last-month-of-life (LML) interview survey	Last month of life	Physical; Spiritual
Sharpe et al. (2005)	Partially	(3) Sufficiency of service provision to resolve symptoms and concerns	Felt	Patient and family members’ reports of symptoms or concerns and if these were resolved by care provision	Supportive Care Needs Survey (36-item interview schedule)	Multiple timepoints (T1=time of interview, T2=3 months T3=6 months)	Psychological; Spiritual; Practical; Information; Physical; Financial/legal; Social
Vogt et al. (2021)	No	(3) Sufficiency of service provision to resolve symptoms and concerns	Felt	Patient reports of symptoms or concerns and if these were resolved by care provision	Modified version of Supportive Care Needs Survey - Short Form (SCNS-SF34)	Past month	Physical; Psychological; Information; Healthcare; Sexuality
Retrospective cohort studies							
Arabadzhyan et al. (2024)	Partially	(2) Access to services	Normative	Routine data including diagnosis and care service use	Medical records	Last month of life	Healthcare
Axelsson et al. (2018)	No	(1) Symptoms and concerns	Normative	Healthcare staff reports of unresolved symptoms	Swedish Register of Palliative care	Last week of life	Physical; Psychological; Information
Hildenbrand et al. (2022)	No	(1) Symptoms and concerns (2) Access to services	Felt	Patient reports of symptoms and concerns; care service use data	Distress Thermometer (DT)	No time frame stated for unmet need measurement	Physical; Psychological; Spiritual; Practical; Social
Hwang et al. (2019)	No	(2) Access to services	Normative	Routine data on care service use	Medical records	No time frame stated for unmet need measurement	Healthcare
Safabakhsh et al. (2023)	Partially	(2) Access to services	Normative	Routine data including diagnosis and care service use	Medical records	Previous 2 years	Physical; Healthcare
Westley-Wise et al. (2022)	No	(2) Access to services	Normative	Routine data including diagnosis, clinical characteristics and care service use	Medical records	Last year of life	Healthcare
Experimental study							
Paterson et al. (2018)	Partially	(3) Sufficiency of service provision to resolve symptoms and concerns	Felt	Patient reports of symptoms or concerns and if these were resolved by care provision	Supportive Care Needs Survey - Short Form (SCNS-SF34)	Past month	Physical; Psychological; Information; Healthcare; Sexuality
Mixed-methods studies							
Paterson et al.(2017)	No	(3) Sufficiency of service provision to resolve symptoms and concerns	Felt	Patient reports of symptoms or concerns and if these were resolved by care provision	Supportive Care Needs Survey - Short Form (SCNS-SF34)	Past month	Physical; Psychological; Information; Healthcare; Sexuality
Spence et al. (2020)	No	(1) Symptoms and concerns (2) Access to services	Felt	Patients reports of symptoms or concerns; care service use data	Edmonton Symptom Assessment System Revised (ESAS-r); Canadian Problem Checklist (CPC)	At the time of measurement	Physical; Psychological; Spiritual; Practical; Overall wellbeing; Social; Information
Teno et al. (2021)	Partially	(3) Sufficiency of service provision to resolve symptoms and concerns	Felt	Family members’ reports of symptoms or concerns and if these were resolved by care provision	Unnamed questionnaire	Last month of life	Physical; Psychological; Spiritual; Information
Cross-sectional studies							
Abu-Odah et al. (2022)	Yes (“*Unmet needs in patients refer to the gap between a patient’s need or expectations for those services and the actual experience of receiving them*.”)	(3) Sufficiency of service provision to resolve symptoms and concerns	Felt	Patient reports of symptoms or concerns and if these were resolved by care provision	Supportive Care Needs Survey - Short Form (SCNS-SF34)	Past month	Physical; Psychological/emotional; Spiritual; Informative; Mobility/functional; Other: patient care and support and sexuality
Alnajar et al. (2024)	Partially	(3) Sufficiency of service provision to resolve symptoms and concerns	Felt; Comparative	Patient reports of symptoms or concerns and if these were resolved by care provision	Problems and Needs in Palliative Care - short version (PNPC-sv)	No time frame stated for unmet need measurement	Physical; Psychological/emotional; Communicative; Spiritual; Practical; Informative; Mobility/functional; Care service use; Other: social, financial, autonomy
Anderson et al. (2001)	Partially	(3) Sufficiency of service provision to resolve symptoms and concerns	Felt; Normative; Comparative	Patient and healthcare staff reports of symptoms or concerns, care service use, and if care resolved symptoms	Unnamed questionnaire	No time frame stated for unmet need measurement	"Physical; Psychological/emotional; Communicative; Mobility/functional; Care service use; Other: Family support
Aranda et al. (2005)	Yes (“*Unmet needs are defined as ‘the requirement of some action or resource* that is *necessary, desirable or useful to attain optimal well-being’*.”)	(3) Sufficiency of service provision to resolve symptoms and concerns	Felt	Patient reports of symptoms or concerns and perceived care needs	Supportive Care Needs Questionnaire (SCNQ)	Past month	Physical; Psychological/emotional; Communicative; Practical; Mobility/functional; Care service use; Other: Patient care and support and sexuality.
Blindbaek et al. (2014)	No	(3) Sufficiency of service provision to resolve symptoms and concerns	Felt	Patient reports of symptoms or concerns and if these were resolved by care provision	Three Levels of Needs Questionnaire (3LNQ)	Past month	Physical; Psychological/emotional; Other: Social, work performance, sexuality
Buzgova et al. (2014)	No	(3) Sufficiency of service provision to resolve symptoms and concerns	Felt	Patient reports of symptoms or concerns and if these were resolved by care provision	Patient Needs Assessment in Palliative Care (PNAP)	No time frame stated for unmet need measurement	Physical; Psychological/emotional; Spiritual; Other: Autonomy, Social
Buzgova et al.^c^ (2016)	Partially	(3) Sufficiency of service provision to resolve symptoms and concerns	Felt	Patient reports of symptoms or concerns and if these were resolved by care provision	Patient Needs Assessment in Palliative Care (PNAP)	No time frame stated for unmet need measurement	Physical; Psychological/emotional; Spiritual; Informative; Other: Social realm, Respect and support, Autonomy,
Chuang et al. (2017)	Partially	(3) Sufficiency of service provision to resolve symptoms and concerns	Normative	Routine data including diagnosis and care service use	Medical records	No time frame stated for unmet need measurement	Care service use
Connor et al. (2005)	No	(3) Sufficiency of service provision to resolve symptoms and concerns	Felt	Family members’ reports of symptom and concerns and if these were resolved by care provision	Family Evaluation of Hospice Care (FEHC) survey	No time frame stated for unmet need measurement	Physical; Psychological
Cooper et al. (2021)	Partially	(2) Access to services	Normative	Routine data (diagnosis)	Medical records	No time frame stated for unmet need measurement	Physical; Psychological; Information; Practical; Healthcare
Cooper et al. (2024)	Partially	(2) Access to services	Normative	Routine data including diagnosis and service use	Medical records	No time frame stated for unmet need measurement	Healthcare
Currow et al. (2008)	Yes (“*Those who received* [specialist palliative care] *services whose needs may have been adequately met by their existing health service providers; and those who did not receive services but may have benefited from accessing them. This approach requires knowledge of numerators (those seen and not seen currently) and the denominator (all people with a life-limiting illness)*.”)	(2) Access to services	Felt; Normative	Family members’ reports of service use and quality of or satisfaction with care	South Australian Health Omnibus Survey	No time frame stated for unmet need measurement	Healthcare
DeGroot et al. (2023)	Partially	(1) Symptoms and concerns	Felt	Patient reports of symptoms or concerns	Integrated Palliative Care Outcome Scale (IPOS)	Last week of life	Physical; Psychological; Information; Practical
Driessen et al. (2023)	Partially	(3) Sufficiency of service provision to resolve symptoms and concerns	Felt	Patient reports of symptoms or concerns and if these were resolved by care provision	Problems and Needs in Palliative Care - short version (PNPC-sv)	No time frame stated for unmet need measurement	Physical; Psychological; Spiritual; Practical; Autonomy; Social; Financial/legal
Effendy et al. (2015)	No	(3) Sufficiency of service provision to resolve symptoms and concerns	Felt	Patient reports of symptoms or concerns and if these were resolved by care provision	Problems and Needs in Palliative Care - short version (PNPC-sv)	No time frame stated for unmet need measurement	Physical; Psychological; Spiritual; Autonomy; Financial/legal
Hasegawa et al. (2016)	Partially	(3) Sufficiency of service provision to resolve symptoms and concerns	Felt	Patient reports of symptoms or concerns and if these were resolved by care provision	Supportive Care Needs Survey - Short Form (SCNS-SF34)	Past month	Physical; Psychological; Information; Healthcare; Sexuality
Hasegawa et al. (2021)	No	(2) Access to services	Felt	Family members’ reports of service use	Unnamed questionnaire	No time frame stated for unmet need measurement	Physical; Practical
Huang et al. (2020)	No	(3) Sufficiency of service provision to resolve symptoms and concerns	Felt	Patient reports of symptoms or concerns and if these were resolved by care provision	Supportive Care Needs Survey - Short Form (SCNS-SF34)	Past 2–6 months	Physical; Psychological; Information; Healthcare; Sexuality
Husain et al. (2013)	No	(3) Sufficiency of service provision to resolve symptoms and concerns	Felt	Patient reports of symptoms or concerns and if these were resolved by care provision	Supportive Care Needs Survey - Short Form (SCNS-SF34)	Past month	Physical; Psychological; Information; Healthcare; Sexuality
Hwang et al. (2004)	No	(3) Sufficiency of service provision to resolve symptoms and concerns	Felt	Patient reports of symptoms or concerns and if these were resolved by care provision	Unnamed questionnaire	Last month of life	Physical; Psychological; Financial/legal; Healthcare; Social
Jeyasingam et al. (2008)	Partially	(3) Sufficiency of service provision to resolve symptoms and concerns	Felt	Patients and family members’ reports of symptoms or concerns related to activities of daily living	Screening Tool Activities of Daily Living (ST-ADL)	No time frame stated for unmet need measurement	Physical
Johnsen et al. (2013)	Partially	(3) Sufficiency of service provision to resolve symptoms and concerns	Felt	Patient reports of symptoms or concerns and if these were resolved by care provision	Three Levels of Needs Questionnaire (3LNQ)	Past week	Physical; Psychological; Practical; Social; Sexuality
Kavalieratos et al. (2014)	No	(1) Symptoms and concerns (2) Access to services	Felt; Comparative	Patient reports of unresolved symptoms; care service use data	McCorkle Symptom Distress Scale; Medical records	No time frame stated for unmet need measurement	Physical; Psychological
Khan et al. (2012)	No	(3) Sufficiency of service provision to resolve symptoms and concerns	Felt	Patient and family members’ reports of symptoms or concerns and if these were resolved by care provision	Problems and Needs in Palliative Care - short version (PNPC-sv)	No time frame stated for unmet need measurement	Physical; Psychological; Spiritual; Information; Autonomy; Social; Financial/legal
Miniotti et al. (2019)	No	(3) Sufficiency of service provision to resolve symptoms and concerns	Felt	Patient reports of symptoms or concerns, sufficiency of care and desire for further attention	Supportive Care Needs Survey - Short Form (SCNS-SF34)	Past month	Physical; Psychological; Practical; Information; Sexuality
Munn et al. (2006)	No	(3) Sufficiency of service provision to resolve symptoms and concerns	Felt	Family members’ and healthcare staff reports of personal care needs and if these were resolved by care provision	Unnamed questionnaire	No time frame stated for unmet need measurement	Physical
Oh et al. (2019)	No	(3) Sufficiency of service provision to resolve symptoms and concerns	Felt	Patient reports of supportive care needs that were not resolved	Amyotrophic Lateral Sclerosis Supportive Care Needs Instrument (ALSSCN)	Past month	Physical; Psychological; Spiritual; Practical; Information; Social
Osse et al. (2005)	Partially	(3) Sufficiency of service provision to resolve symptoms and concerns	Felt	Patient reports of symptoms or concerns and if these were resolved by care provision	Problems and Needs in Palliative Care (PNPC)	No time frame stated for unmet need measurement	Physical; Psychological; Spiritual; Role activities; Financial/legal; Social; Autonomy; Healthcare
Park et al. (2017)	Partially	(3) Sufficiency of service provision to resolve symptoms and concerns	Felt	Patient reports of spiritual needs and whether these were met	Unnamed questionnaire	No time frame stated for unmet need measurement	Spiritual
Pearce et al. (2012)	Partially	(3) Sufficiency of service provision to resolve symptoms and concerns	Felt	Patient reports of spiritual needs and whether these were met	Unnamed questionnaire	No time frame stated for unmet need measurement	Spiritual
Rachakonda et al. (2015)	Yes (“*there is a discernible, often ignored under-evaluated care-management gap in palliative cancer care, where the estimated clinical outcome is seldom translated into a patient-centered benefit. These supportive care needs that call for immediate attention are classified as ’unmet needs'*”)	(3) Sufficiency of service provision to resolve symptoms and concerns	Felt	Patient reports of symptoms or concerns and their continued needs for care	Needs Assessment for Advanced Cancer Patients (NA-ACP)	No time frame stated for unmet need measurement	Physical; Psychological; Spiritual; Information; Financial/legal; Social
Rhodes et al. (2012)	No	(3) Sufficiency of service provision to resolve symptoms and concerns	Felt	Family members’ reports of how well care provided matched the decedent’s and family’s needs	Family Evaluation of Hospice Care (FEHC) survey	No time frame stated for unmet need measurement	Physical; Psychological; Spiritual
Schenker et al. (2014)	No	(3) Sufficiency of service provision to resolve symptoms and concerns	Felt	Patient reports of symptoms or concerns and if these were resolved by care provision	Unnamed questionnaire	Past month	Physical; Psychological; Information; Spiritual; Social
Strupp et al. (2018)	No	(1) Symptoms and concerns	Felt	Patient reports of symptoms or concerns and their care needs in relation to how severely affected they are by their disease	Unnamed questionnaire	No time frame stated for unmet need measurement	Open question about aspects in which the patient wishes for more help or support
Szekendi et al. (2016)	Partially	(1) Symptoms and concerns; (2) Access to services	Normative	Routine data including diagnosis and care service use	Medical records	No time frame stated for unmet need measurement	Physical
Teno et al. (2004)	No	(3) Sufficiency of service provision to resolve symptoms and concerns	Felt	Family members’ reports of symptoms or concerns and if these were resolved by care provision	Structured interview	Final 3 days of life or less	Physical; Psychological
Teno et al. (2007)	No	(3) Sufficiency of service provision to resolve symptoms and concerns	Felt	Family members’ reports of symptoms or concerns and if these were resolved by care provision	Family Evaluation of Hospice Care (FEHC) survey	No time frame stated for unmet need measurement	Physical; Psychological
Teno et al. (2011)	No	(3) Sufficiency of service provision to resolve symptoms and concerns	Felt	Family members’ reports of symptoms or concerns and if these were resolved by care provision	Family Evaluation of Hospice Care (FEHC) survey	No time frame stated for unmet need measurement	Physical; Autonomy
Trandel et al. (2019)	No	(3) Sufficiency of service provision to resolve symptoms and concerns	Felt	Patient reports of symptoms or concerns and if these were resolved by care provision	Supportive Care Needs Survey - Short Form (SCNS-SF34)	Past month	Physical; Psychological; Information; Healthcare; Sexuality
Wang et al. (2021)	No	(3) Sufficiency of service provision to resolve symptoms and concerns	Felt	Patient and family members’ reports of symptoms or concerns and if these were resolved by care provision	Problems and Needs in Palliative Care - short version (PNPC-sv)	No time frame stated for unmet need measurement	Physical; Psychological; Information; Spiritual; Practical; Autonomy; Social; Financial/legal
Wang et al. (2023)	No	(1) Symptoms and concerns	Felt	Patient reports of symptoms or concerns	Palliative Outcome Scale (POS)	Past week	Physical; Psychological; Spiritual; Information; Practical
Watson et al. (2019)	No	(3) Sufficiency of service provision to resolve symptoms and concerns	Felt	Patient reports of symptoms or concerns and if these were resolved by care provision	Modified version of Supportive Care Needs Survey - Short Form (SCNS-SF34)	Past month	Physical; Psychological; Information; Healthcare; Sexuality; Financial/legal
Wegier et al. (2021)	Partially	(1) Symptoms and concerns	Felt	Patient and family members’ reports of symptoms or concerns	Edmonton Symptom Assessment Scale Revised (ESAS-r); Sudore’s Advance Care Planning (ACP) Engagement Survey	At the time of measurement	Physical; Psychological; Information
Systematic reviews							
Bore et al. (2024)	Yes (“*Unmet needs for supportive care services represent the gap between a cancer patient’s desire or need for specific services and their received experiences*.”)	Not applicable due to no direct measurement of unmet palliative care needs	Felt				
Chen et al. (2020)	No		Felt				
Fu et al. (2020)	Yes (“*Unmet supportive care needs reflect the disparity between the supports that an individual perceives as necessary and those that are actually provided*.”)		Felt				
Harrison et al. (2009)	Yes (“*Needs that were not addressed and where additional support was required were classified as ‘unmet needs’ . . . Unmet needs assessment adds a further dimension to needs assessment by distinguishing how well needs have been met and identifying those that remain unmet.*”)		Felt				
Hart et al. (2022)	No		Felt				
Moghaddam et al. (2016)	Yes (“*In the context of supportive care, unmet needs reflect incongruity between the supports that an individual perceives to be necessary* versus *the actual supports provided*.”)		Felt				
Schmidt et al. (2023)	Yes (“*The definition of unmet healthcare needs was constructed stepwise*, first *defining healthcare needs as what patients and the population as a whole desire to receive from healthcare services to improve overall health. Unmet needs were defined as needs that are either not addressed or receive insufficient attention.*”)		Felt				
Ventura et al. (2014)	Yes (“*Needs go unmet when basic requirements to maintain quality of life have not been met. For patients, unmet needs tend to exist across practical, emotional, physical and existential domains.*”)		Felt				
Wang et al. (2018)	Yes (“*Unmet needs assessment is designed to identify how well and how much their needs have been satisfied or not.*”)		Felt				

a Study also reported in Halkett et al. (2015).^103^

b Study also reported in Kirkland et al.^18^ and Kruhlak et al. (2021).^104^

c Study also reported in & Sikorova (2015).^105^

* To increase readability, we do not denote studies using the third approach as also using the first approach, even though both include measurement of symptoms and concerns. In the third approach, symptom measurement is part of the process of measuring unmet palliative care needs and not indicative of unmet needs in itself (as it is in approach 1).

‡ Domains based on World Health Organization^24^ framework of comprehensive needs assessment in palliative care: Physical (key indicators include pain, breathlessness, function/daily living activities), Psychological (Depression, anxiety, isolation), Spiritual (meaning of life, acceptance of dying, religious support), Social (support from social network, maintaining relations, express feelings with others), Information (information about diagnosis, prognosis and care options), Financial/legal (financial concerns, handling financial and legal arrangements, e.g. wills), Practical (ability to maintain personal hygiene, household tasks), Autonomy (dependency on others, experiencing loss of control, privacy), Role activities (difficulties with employment or studies, difficulty caring for children), Personal issues (handling personal affairs), and Healthcare (support from healthcare professionals, side effects, quality of care), as well as the added domain “Sexuality” (sexual dysfunction, loss of libido).

Definitions varied but often emphasised a perceived discrepancy between the care required or desired and the care received, such as “the disparity between the supports that an individual perceives as necessary and those that are actually provided.”^98^ Other definitions highlighted presence of unfulfilled care needs, for example, “needs that are either not addressed or receive insufficient attention.”^101^ Some studies focussed on specific outcomes, for example, conceptualising unmet palliative care needs as lacking “the requirement of some action or resource that is necessary, desirable or useful *to attain optimal well-being*”^38^ or “when basic requirements *to maintain quality of life* have not been met.”^102^

Partial definitions were usually presented as part of the study rationale, to suggest that care needs in specific patient groups or care settings might not be met. One study stated that “problems in the quality of life are not always correctly identified, and needs for care sometimes remain unmet.”^93^ Other studies implied that known issues of under-utilisation of palliative care meant that care needs might not be met, such as arguing that “Deficits in [palliative] care in any domains may result in multidimensional, unmet needs”.^48^

Where studies specified care services that can help address unmet palliative care needs in their definition, few were explicit about which these services were or made any distinction between specialist and generalist palliative care.

## Measurement of unmet palliative care needs

### Approaches to measurement

While few studies defined unmet palliative care needs, most described the approach used to measure it (i.e. the operationalisation), whether explicitly or implicitly. We identified three main approaches to measuring unmet palliative care needs: (1) *Symptoms and concerns* (studies using symptom prevalence to indicate unmet palliative care needs, without explicitly determining whether these symptoms were addressed or not); (2) *Access to services* (studies focussing on measuring service referrals or contacts, with little consideration of care outcomes); and (3) *Sufficiency of service provision to resolve symptoms and concerns* (studies addressing a perceived gap between the care provided and care deemed necessary to resolve symptoms and concerns). These approaches were not mutually exclusive and occasionally combined to varying degrees. Examples of each approach are presented in Box 1.

**Box 1.** Examples of approaches to measuring unmet palliative care needs in the included studies.

Approach to measurement	Example of approach as used in the included studies
Symptoms and concerns	*Unresolved palliative care needs were defined as having* [Integrated Palliative care Outcome Scale (IPOS)] *symptoms scores above 2* [i.e. being moderately affected by a symptom or concern].
	Hamano et al.^52^
Access to services	*Unmet need was estimated as the difference in the proportion of decedents who were estimated to have needed palliative care* [according to 10 pre-determined conditions] *and those who accessed relevant services* [admission to designated palliative care units or an episode of care under a palliative care specialist in any other setting].
	Westley-Wise et al.^92^
Sufficiency of service provision to address symptoms and concerns	*Unmet need was indicated when the patient responded that they experienced a problem either very much or to some extent and would have found additional assistance very or somewhat helpful*.
	Hwang et al.^60^
	*A need was defined as unmet when the respondent reported that the patient did not receive any or not enough help with that symptom.*
	Teno et al.^84^

#### Symptoms and concerns

The first approach to measurement of unmet palliative care needs relates to identifying and reporting the presence of one or more symptoms or concerns. Descriptions of what constituted a symptom or concern varied. Some studies narrowly focussed on certain levels of specific symptoms, for example, prevalence of pain being operationalised as having unmet need for pain relief.^39^ Other studies acknowledged accumulation of several symptoms or concerns, or these being present across various domains as indicating unmet needs,^48,52^ while others included anything that the patient deemed a problem or concern.^81^ Studies focussing on symptoms and concerns were often based on self- or proxy- reports of felt needs (in contrast to normative needs).

#### Access to services

The second approach to measurement of unmet palliative care needs relates to the access to or provision of care. This includes absence of referral to specific services (such as specialist palliative care) for people who might benefit from this care, for example, due to having specific diagnoses or other clinical characteristics. Access to palliative services (whether generalist or specialist) was generally presumed to meet needs and often described in terms of maintaining or improving quality of life, whereas lack of contact with palliative services was indicative of unmet needs. These studies were often larger-scale studies using medical records^78^ or clinical screening tools, for example, the Palliative Care and Rapid Emergency Screening (P-CaRES) tool,^66^ to make inferences about people missing out on palliative care receipt at cohort- or population- level. As such these largely represent normative needs, that is, as determined by professionals.^12^ This approach seldom distinguished between types of palliative care services, for example, studies reporting overall rates of palliative care referrals.^45,47,82^ Availability of palliative care services was generally assumed, with only one study explicitly considering the lack of services across geographical regions.^80^

#### Sufficiency of service provision to resolve symptoms and concerns

Most studies’ approaches to measuring unmet palliative care needs related to insufficient care provision. This included the presence of symptoms, concerns or problems that were either not addressed at all, not adequately addressed (according to the patient or their family), or not satisfactorily resolved (for which the patient wanted further professional attention). This approach emphasises a gap or discrepancy between a desired or expected standard of care and what has been provided. Studies often measured insufficient provision of care by asking patients or proxies (usually patients’ families or, less commonly, healthcare professionals) first whether a problem or concern was present and then the extent to which it was adequately provided for and addressed, thus primarily capturing felt need.^12^ This approach was primarily used by small-scale survey studies using convenience samples to describe patients’ perspectives on the presence of unresolved issues, although some were larger scale.^56,84^

### Sources of information for measuring unmet palliative care needs in primary evidence studies

Among primary evidence studies (n = 61) that measured unmet needs, methods for measurement varied in terms of who and what was the source of information for the assessment (see Table 3). Most studies used self-reported data (*n* = 39, 68%) collected using research questionnaires or patient reported or patient-centred outcome measures (PROMs or PCOMs). Proxy-reported data by bereaved family members, for example, using mortality follow-back surveys were also common (*n* = 15, 25%). These methods largely capture felt needs (i.e. an individual’s perceived need for care). Clinical assessments by healthcare professionals (often through use of screening tools; *n* = 8, 11%) or researchers (*n* = 8, 11%) were less common, representing normative need.^12^

### Instruments and timing of measurement in primary evidence studies

As shown in Table 3, named instruments or measures were used in 42 (69%) of the primary evidence studies, whereas nine (15%) used unnamed questionnaires, and one (2%) used an unnamed structured interview. The most commonly used named measure was the Supportive Care Needs Survey - Short Form (SCNS-SF34; *n* = 8, 13%), followed by the Problems and Needs in Palliative Care-short version (PNPC-sv; *n* = 5, 8%), both of which were initially developed for cancer patients. Unmet palliative care needs were estimated using administrative medical records in nine (15%) studies.

Almost half of the studies did not report the time period during which unmet palliative care needs were measured (*n* = 30, 49%). Of those that specified a time interval, self-reported unmet needs commonly related to the past month (*n* = 15, 25%). In studies using samples of decedents, the time interval of interest was usually the last month of life (*n* = 4, 7%) or last week of life (*n* = 3, 5%). Only three studies reported longitudinal measurements of unmet palliative care needs over time.^52,88,94^

### Reporting of unmet palliative care needs in primary evidence studies

Some of the 61 primary evidence studies reported unmet palliative care needs at the item-level, usually as the proportion of patients with a specific unresolved symptom or concern.^39,44,85^ Most, however, reported unmet needs at the domain-level by combining responses to several items related to a specific domain of care (e.g. Oh et al.^70^ and Pearce et al.^74^). Our mapping of the unmet care needs reported in the primary evidence captured 11 distinct domains of needs (Table 3). Ten of these were identified in the framework by Goni-Fuste et al.,^32^ with the addition of “Sexuality” which was reported in 14 (23%) studies. Of the 52 primary evidence studies that measured unmet palliative needs using a questionnaire or interview, most used multi-domain measures that allowed a holistic assessment of unmet palliative care needs: 38 (73%) captured three dimensions or more, while only four (8%) focussed solely on one domain (spiritual needs^53,71,74^ and physical function^61^).

In total, 26 studies (43%) reported one single metric of unmet palliative care needs, usually as a percentage of participants needing palliative care but not adequately receiving it. These rates ranged from 10% in a 2023 study of hospitalised patients in United States^78^ to 30% among people with non-cancer conditions in a 2008 health omnibus survey in Australia.^47^ Other studies calculated unmet palliative care needs by aggregating items and/or domains from the measure used, which was commonly reported as prevalence of having one or more unmet needs (e.g. “71.8% of patients reported at least one unmet supportive care need”^88^), sometimes with reference to severity (e.g. “48% reported one or more moderate or high unmet needs”^91^). Some studies presented a mean or median number of unmet palliative care needs within the group (e.g. “The median number of total unmet needs identified was 3”^79^).

## Discussion

### Key findings

This scoping review examines how existing evidence has defined, measured and reported unmet palliative care needs across disease groups and care settings. Only 11 out of 70 (16%) defined the theoretical construct of unmet palliative care needs. The review also found variability in approaches to measurement and reporting across studies, identifying three main approaches to measuring unmet palliative care needs: (1) the presence or severity of symptoms and concerns; (2) the level of access to palliative care services; and (3) symptoms and concerns that remain unresolved despite service provision.

Unmet palliative care needs were commonly identified using self-reported data about the presence of symptoms and concerns and whether these were resolved by the care provided. Similar to previous reviews on unmet palliative care needs in the emergency department^106^ and in nursing homes,^107^ we found that the lack of explicit definition hinders consistent measurement and comparisons across studies, making it difficult to determine how best to measure or estimate unmet needs at the population-level. This lack of clarity creates ambiguity about what is being assessed and risks conflating unmet palliative care needs with related concepts, such as satisfaction with care or quality of life. There is also a conceptual distinction between unmet palliative care needs as outcomes (e.g. inadequate pain relief), and reasons for unmet needs (e.g. not accessing to palliative care services), which was rarely noted in the included studies.

Expanding on the findings of Franks et al.^15^ on population-level assessments of palliative care needs, our review identifies three approaches to measuring *unmet* needs in the existing international literature.

The first approach, *Symptoms and concerns*, involves quantifying prevalence or burden of problems. This approach corresponds to Franks et al.^15^ epidemiological approach and is not dependent on whether patients are known to services or not. Patient-reported or patient-centred outcome measures (PROMs and PCOMs) can be very helpful for collecting these data and, depending on how many domains a measure captures, this approach enables a holistic assessment of palliative care needs. For example, the Integrated Palliative care Outcome Scale (IPOS) is a valid and reliable measure for multi-domain symptoms and concerns, allowing evaluation of the complexity of needs for care.^108^ Wide international use of PROMs and increasing integration with patient record systems further aids access to comparable data for research. Nevertheless, this approach has limitations, as presence of unresolved symptoms or concerns does not necessarily mean professional help is desired, and it may not clarify which professionals or services should meet any unmet needs identified.

The second approach, *Access to services*, infers unmet palliative care needs from access to services, aligning with Franks et al.’s^15^ healthcare utilisation approach. Service use is usually documented in medical records and often readily available in national patient registers, which facilitates their use in research, policymaking, and commissioning. While evaluating access to services, for example, referrals, can elucidate care gaps or inequities indicative of underserved populations, this approach requires that patients are correctly identified as having palliative care needs in the first place.^109^ Moreover, uptake of palliative care services might not reflect actual needs for care,^47^ and access to services alone cannot indicate whether needs are met or if the care delivered improves the patient’s situation.^95^

The third approach identified in this review, *Sufficiency of service provision to resolve symptoms and concerns*, is the most comprehensive as it combines aspects of the first two in operationalising unmet palliative care needs (presence of symptoms and concerns but also whether services adequately addressed these). This approach is also the only that captures forgone care. Studies using this approach more often distinguished between having an unmet problem and desire for care, showing that patients did not necessarily want professional attention even for serious problems, especially within social, sexual, and spiritual domains.^62,74,93^ This approach is helpful for evaluating outcomes of care and person-centredness,^110^ that is, not only determining whether symptoms are present or if care is delivered, but whether the care meets individual needs. However, focussing on the gap between desired and received care can risk overestimating unmet palliative care needs if expectations of symptom relief at the end of life are unrealistic. For example, a symptom like fatigue^111^ is known to be common towards the end of life but difficult to fully relieve even with optimal currently available treatment. Studies adopting this approach must consider the subjective nature of measurement and how the person making the assessment might influence estimations of unmet need, since reports of symptom relief can vary between family members and healthcare professionals.^112^

Each approach has its strengths and limitations, and all can help estimating unmet palliative care needs, depending on context and research question. For example, the *Symptoms and concerns* approach is well suited for intervention studies to assess changes in needs over time in response to a care intervention. The *Access to services* approach is helpful for service mapping studies, and the *Sufficiency of service provision to resolve symptoms and concerns* approach could be used to inform future post-bereavement survey research. Appropriateness of the approaches may also vary according to the healthcare system from which the evidence is derived.^113^ As already mentioned, the approaches do not distinguish between which services should meet which level of unmet palliative care needs. Future research should examine how variation in service use might be associated with management of unmet needs across the physical, psychological, spiritual and social domains. The high proportions of unmet palliative care needs reported in the included studies also raise questions about “acceptable” levels of unmet needs at the population-level. While the goal should be to prevent suffering, achieving complete resolution of all symptoms and concerns for every individual is unlikely given finite care resources. Some degree of unmet needs may therefore be inevitable. The definitions of unmet palliative care needs presented in this review cover a wide span, ranging from care required for optimal well-being^38^ to basic requirements for maintaining quality of life,^102^ and provide little clarity about what constitutes a feasible standard.

### What this review adds

Resource limitations invariably mean that specialist palliative care access is prioritised for the most urgent and complex cases,^114^ and it is not surprising that the existing research has focussed on identifying specialist palliative care needs. However, better understanding of whether specialist palliative care services meet patients’ needs is crucial to inform decision-making about funding, planning and delivering high-quality care.^115^ Despite 25 years since Franks et al.’s^15^ review highlighted poor-quality and inconsistent population-level estimates of palliative care needs, the literature is still largely based on cross-sectional studies using self-reported data from convenience samples, with few studies using population-based or representative samples.

To provide a comprehensive understanding of unmet palliative care needs, multi-domain multi-level assessments like those presented in the third approach to measurement are well-suited to capture both felt and normative need, particularly when combining objective data sources, like service provision, and subjective experiences of the care received. In Box 2 we provide further recommendations for advancing research, policy and practice and building a more robust evidence-base for estimating the prevalence of unmet palliative care needs in the general population. Additional considerations of data collection methods are provided in Supplemental File 3.

**Box 2.** General and audience-specific recommendations based on the review findings.

Audience	Recommendation(s)
General	- Explicit reporting of definition of unmet palliative care needs (e.g., as representing the gap between required and received care for people with life-limiting illness to manage multi-domain symptoms and concerns) and align definitions with three approaches to measurement: *Symptoms and concerns; Access to services*; or *Sufficiency of service provision to resolve symptoms and concerns*.
Researchers	- Future studies should adopt the above definition and specify their approach to measure unmet palliative care needs and consider care receipt from different services in the analyses.
	- Research is needed into which needs are best met by generalist and/or specialist palliative care services.
	- Use of frameworks such as Guidance for Reporting Involvement of Patients and the Public^116^ to increase transparency of the development and validation processes of measures used.
Policy makers	- Provide guidance on how to assess unmet palliative care need for commissioners, underpinned by evidence.
	- Implement policies that support the use of routine collection of information about unmet palliative care need at local and national levels, for example a regular mortality follow-back survey.
Commissioners	- Ensure that clinical needs assessments of patients include identification of *unmet* palliative care needs and use this to inform local service planning.
Service managers & practitioners	- Training for healthcare professionals on using validated screening tools or self-report measures to identify unmet palliative care needs.
	- Use regular holistic assessments of symptoms and concerns to identify unmet palliative care needs and inform service delivery.

Routine data collection from continuous multi-domain needs assessments is crucial to advance the understanding of unmet palliative care needs at the population-level and inform policymakers and commissioners in the planning and delivery of palliative and end-of-life care.^23,117^ For studies using administrative data from medical records, standardising the use of clinical variables in screening tools would enhance consistency in identification of unmet palliative care needs. Challenges remain, however, regarding how needs are disclosed, as limited awareness of available services or resources can hinder people from recognising they have unmet palliative care needs,^118^ and lack of trust and power imbalances can hinder requests for support, particularly for potentially sensitive topics such as mental health issues, living situations, or family relationships.^79^

### Strengths and limitations

This is the first scoping review to summarise how unmet palliative care needs has been defined, measured and reported in existing literature. While prior reviews have focussed on the psychometric properties of measures of unmet palliative care needs,^18,19,119^ this review contributes with new knowledge about overarching theoretical and operational aspects of the existing evidence on unmet palliative care needs.

A strength of this review is the inclusion of grey literature as well as use of forward and backward citation searches to mitigate the risk of omitting relevant sources. We present data at the study-level, thereby avoiding double counting duplicate data sets across sources.^29^ Although the methodological variation of included studies hindered conclusions to be drawn regarding prevalence of unmet palliative care needs, this is not a limitation for the current scoping review since the aim was to identify and describe the breadth of existing evidence and not synthesise study findings.^120^

By not limiting our inclusion criteria to specific disease groups or care settings, we provide a comprehensive overview of how unmet palliative care needs has been researched in various contexts, regardless of diagnosis, with the objective to expand on the work of Franks et al.^15^ and further the understanding of how prevalence of unmet palliative needs are measured. However, our review also has limitations. Including only quantitative or mixed-methods studies and reviews reduces the range of evidence sources and may limit the completeness of the findings, as we might have missed important conceptual work from qualitative sources. The lack of quality appraisal and risk of bias assessment is another inherent limitation to scoping reviews,^121^ but also allows for a more rapid review process.

## Conclusion

This is the first scoping review to summarise the existing literature on how unmet palliative care needs have been defined, measured and reported across disease groups and care settings. Even though quantification of unmet palliative care needs is crucial for planning and equitable delivery of palliative care services, our review demonstrates that studies rarely explicitly define the construct and there is considerable variation in how unmet palliative care needs are measured and reported. Clear reporting of definitions and operationalisations of unmet palliative care needs is required to harmonise the evidence base and subsequently enable estimation of the proportion of people with unmet needs at the population-level. We identify three distinct approaches to measurement of unmet palliative care needs – (1) *symptoms and concerns*; (2) *access to services*; and (3) *sufficiency of service provision to resolve symptoms and concerns* – and provide guidance on their use for various end-users.

## Supplemental Material

sj-docx-1-pmj-10.1177_02692163261416279 – Supplemental material for Defining and measuring unmet palliative care needs among people with life-limiting illness: A scoping review of international evidence Supplemental material, sj-docx-1-pmj-10.1177_02692163261416279 for Defining and measuring unmet palliative care needs among people with life-limiting illness: A scoping review of international evidence by Therese Johansson, Melanie F. J. Diggle, Anne Finucane, Joanna M. Davies, Irene J. Higginson, Katherine E. Sleeman, Lorna K. Fraser, Fliss E. M. Murtagh and Anna E. Bone in Palliative Medicine

sj-docx-2-pmj-10.1177_02692163261416279 – Supplemental material for Defining and measuring unmet palliative care needs among people with life-limiting illness: A scoping review of international evidence Supplemental material, sj-docx-2-pmj-10.1177_02692163261416279 for Defining and measuring unmet palliative care needs among people with life-limiting illness: A scoping review of international evidence by Therese Johansson, Melanie F. J. Diggle, Anne Finucane, Joanna M. Davies, Irene J. Higginson, Katherine E. Sleeman, Lorna K. Fraser, Fliss E. M. Murtagh and Anna E. Bone in Palliative Medicine

sj-docx-3-pmj-10.1177_02692163261416279 – Supplemental material for Defining and measuring unmet palliative care needs among people with life-limiting illness: A scoping review of international evidence Supplemental material, sj-docx-3-pmj-10.1177_02692163261416279 for Defining and measuring unmet palliative care needs among people with life-limiting illness: A scoping review of international evidence by Therese Johansson, Melanie F. J. Diggle, Anne Finucane, Joanna M. Davies, Irene J. Higginson, Katherine E. Sleeman, Lorna K. Fraser, Fliss E. M. Murtagh and Anna E. Bone in Palliative Medicine
