# Supplementary material for: Defining and measuring unmet palliative care needs among people with life-limiting illness: A scoping review of international evidence
Source: Palliat Med. 2026 Feb 6;40(4):448–71. doi: 10.1177/02692163261416279 (PMC13061314; doi:10.1177/02692163261416279)
Supplement: sj-docx-3-pmj-10.1177_02692163261416279 – Supplemental material for Defining and measuring unmet palliative care needs among people with life-limiting illness: A scoping review of international evidence [file sj-docx-3-pmj-10.1177_02692163261416279.docx]

|  | **Unmet needs defined** | **Approach to measurement *** | **Type of need** | **Sources of information for measurement** | **Measure** | **Timing of measurement** | **Domains of need ‡** | |
| --- | --- | --- | --- | --- | --- | --- | --- | --- |
| **Prospective cohort studies** | | | | | | | |  |
| Faris et al.^1^ (2024) | Partially | 3) Sufficiency of service provision to resolve symptoms and concerns | Felt | Patient reports of symptoms or concerns and if these were resolved by care provision | The 9-item SCNS-Screening Tool (SCNS-ST9), a brief version of the 34-item Supportive Care Needs Survey-Short Form (SCNS-SF34) | Past month | Physical; Psychological; Information; Healthcare; Sexuality | |
| Hamano et al. (2023) | Partially | 1) Symptoms and concerns | Felt | Healthcare staff reports of symptoms or concerns | Integrated Palliative Care Outcome Scale (IPOS) | Past month | Physical; Psychological; Information; Practical | |
| Hampton et al. (2007) | No | 3) Sufficiency of service provision to resolve symptoms and concerns | Felt | Patient reports of spiritual needs and whether these were met or not | The Spiritual Needs Inventory (SNI) | No time frame stated for unmet need measurement | Spiritual | |
| Kiely et al. (2010) | No | 3) Sufficiency of service provision to resolve symptoms and concerns | Felt | Family members' reports of symptoms or concerns; care service use data | A modified version of the Toolkit After-Death Bereaved Family Member Interview | Last week of life | Psychological; Information; Practical | |
| Kirkland et al.^2^ (2021) | No | 2) Access to services | Normative | Healthcare staff assessments based on clinical characteristics; care service use data | Medical records | No time frame stated for unmet need measurement | Physical; Healthcare | |
| Makaroun et al. (2018) | No | 3) Sufficiency of service provision to resolve symptoms and concerns | Felt | Family members' reports of symptom or concerns | Last-month-of-life (LML) interview survey | Last month of life | Physical; Spiritual | |
| Sharpe et al. (2005) | Partially | 3) Sufficiency of service provision to resolve symptoms and concerns | Felt | Patient and family members' reports of symptoms or concerns and if these were resolved by care provision | Supportive Care Needs Survey (36-item interview schedule) | Multiple timepoints (T1=time of interview, T2=3 months T3=6 months) | Psychological; Spiritual; Practical; Information; Physical; Financial/legal; Social | |
| Vogt et al. (2021) | No | 3) Sufficiency of service provision to resolve symptoms and concerns | Felt | Patient reports of symptoms or concerns and if these were resolved by care provision | Modified version of Supportive Care Needs Survey - Short Form (SCNS-SF34) | Past month | Physical; Psychological; Information; Healthcare; Sexuality | |
| **Retrospective cohort studies** | | | | | | | |  |
| Arabadzhyan et al. (2024) | Partially | 2) Access to services | Normative | Routine data including diagnosis and care service use | Medical records | Last month of life | Healthcare | |
| Axelsson et al. (2018) | No | 1) Symptoms and concerns | Normative | Healthcare staff reports of unresolved symptoms | Swedish Register of Palliative care | Last week of life | Physical; Psychological; Information | |
| Hildenbrand et al. (2022) | No | 1) Symptoms and concerns  2) Access to services | Felt | Patient reports of symptoms and concerns; care service use data | Distress Thermometer (DT) | No time frame stated for unmet need measurement | Physical; Psychological; Spiritual; Practical; Social | |
| Hwang et al. (2019) | No | 2) Access to services | Normative | Routine data on care service use | Medical records | No time frame stated for unmet need measurement | Healthcare | |
| Safabakhsh et al. (2023) | Partially | 2) Access to services | Normative | Routine data including diagnosis and care service use | Medical records | Previous 2 years | Physical; Healthcare | |
| Westley-Wise et al. (2022) | No | 2) Access to services | Normative | Routine data including diagnosis, clinical characteristics and care service use | Medical records | Last year of life | Healthcare | |
| **Experimental study** | | | | | | | |  |
| Paterson et al. (2018) | Partially | 3) Sufficiency of service provision to resolve symptoms and concerns | Felt | Patient reports of symptoms or concerns and if these were resolved by care provision | Supportive Care Needs Survey - Short Form (SCNS-SF34) | Past month | Physical; Psychological; Information; Healthcare; Sexuality | |
| **Mixed-methods studies** | | | | | | | |  |
| Paterson et al.(2017) | No | 3) Sufficiency of service provision to resolve symptoms and concerns | Felt | Patient reports of symptoms or concerns and if these were resolved by care provision | Supportive Care Needs Survey - Short Form (SCNS-SF34) | Past month | Physical; Psychological; Information; Healthcare; Sexuality | |
| Spence et al. (2020) | No | 1) Symptoms and concerns  2) Access to services | Felt | Patients reports of symptoms or concerns; care service use data | Edmonton Symptom Assessment System Revised (ESAS-r); Canadian Problem Checklist (CPC) | At the time of measurement | Physical; Psychological; Spiritual; Practical; Overall wellbeing; Social; Information | |
| Teno et al. (2021) | Partially | 3) Sufficiency of service provision to resolve symptoms and concerns | Felt | Family members' reports of symptoms or concerns and if these were resolved by care provision | Unnamed questionnaire | Last month of life | Physical; Psychological; Spiritual; Information | |
| **Cross-sectional studies** | | | | | | | |  |
| Abu-Odah et al. (2022) | Yes ("*Unmet needs in patients refer to the gap between a patient’s need or expectations for those services and the actual experience of receiving them*.") | 3) Sufficiency of service provision to resolve symptoms and concerns | Felt | Patient reports of symptoms or concerns and if these were resolved by care provision | Supportive Care Needs Survey - Short Form (SCNS-SF34) | Past month | Physical; Psychological/emotional; Spiritual; Informative; Mobility/functional; Other: patient care and support and sexuality | |
| Alnajar et al. (2024) | Partially | 3) Sufficiency of service provision to resolve symptoms and concerns | Felt; Comparative | Patient reports of symptoms or concerns and if these were resolved by care provision | Problems and Needs in Palliative Care - short version (PNPC-sv) | No time frame stated for unmet need measurement | Physical; Psychological/emotional; Communicative; Spiritual; Practical; Informative; Mobility/functional; Care service use; Other: social, financial, autonomy | |
| Anderson et al. (2001) | Partially | 3) Sufficiency of service provision to resolve symptoms and concerns | Felt; Normative; Comparative | Patient and healthcare staff reports of symptoms or concerns, care service use, and if care resolved symptoms | Unnamed questionnaire | No time frame stated for unmet need measurement | "Physical; Psychological/emotional; Communicative; Mobility/functional; Care service use; Other: Family support | |
| Aranda et al. (2005) | Yes ("*Unmet needs are defined as ‘the requirement of some action or resource that is necessary, desirable or useful to attain optimal well-being’*.") | 3) Sufficiency of service provision to resolve symptoms and concerns | Felt | Patient reports of symptoms or concerns and perceived care needs | Supportive Care Needs Questionnaire (SCNQ) | Past month | Physical; Psychological/emotional; Communicative; Practical; Mobility/functional; Care service use; Other: Patient care and support and sexuality. | |
| Blindbaek et al. (2014) | No | 3) Sufficiency of service provision to resolve symptoms and concerns | Felt | Patient reports of symptoms or concerns and if these were resolved by care provision | Three Levels of Needs Questionnaire (3LNQ) | Past month | Physical; Psychological/emotional; Other: Social, work performance, sexuality | |
| Buzgova et al. (2014) | No | 3) Sufficiency of service provision to resolve symptoms and concerns | Felt | Patient reports of symptoms or concerns and if these were resolved by care provision | Patient Needs Assessment in Palliative Care (PNAP) | No time frame stated for unmet need measurement | Physical; Psychological/emotional; Spiritual; Other: Autonomy, Social | |
| Buzgova et al.^3^ (2016) | Partially | 3) Sufficiency of service provision to resolve symptoms and concerns | Felt | Patient reports of symptoms or concerns and if these were resolved by care provision | Patient Needs Assessment in Palliative Care (PNAP) | No time frame stated for unmet need measurement | Physical; Psychological/emotional; Spiritual; Informative; Other: Social realm, Respect and support, Autonomy, | |
| Chuang et al. (2017) | Partially | 3) Sufficiency of service provision to resolve symptoms and concerns | Normative | Routine data including diagnosis and care service use | Medical records | No time frame stated for unmet need measurement | Care service use | |
| Connor et al. (2005) | No | 3) Sufficiency of service provision to resolve symptoms and concerns | Felt | Family members' reports of symptom and concerns and if these were resolved by care provision | Family Evaluation of Hospice Care (FEHC) survey | No time frame stated for unmet need measurement | Physical; Psychological | |
| Cooper et al. (2021) | Partially | 2) Access to services | Normative | Routine data (diagnosis) | Medical records | No time frame stated for unmet need measurement | Physical; Psychological; Information; Practical; Healthcare | |
| Cooper et al. (2024) | Partially | 2) Access to services | Normative | Routine data including diagnosis and service use | Medical records | No time frame stated for unmet need measurement | Healthcare | |
| Currow et al. (2008) | Yes ("*Those who received* [specialist palliative care] *services whose needs may have been adequately met by their existing health service providers; and those who did not receive services but may have benefited from accessing them. This approach requires knowledge of numerators (those seen and not seen currently) and the denominator (all people with a life-limiting illness)*.") | 2) Access to services | Felt; Normative | Family members' reports of service use and quality of or satisfaction with care | South Australian Health Omnibus Survey | No time frame stated for unmet need measurement | Healthcare | |
| DeGroot et al. (2023) | Partially | 1) Symptoms and concerns | Felt | Patient reports of symptoms or concerns | Integrated Palliative Care Outcome Scale (IPOS) | Last week of life | Physical; Psychological; Information; Practical | |
| Driessen et al. (2023) | Partially | 3) Sufficiency of service provision to resolve symptoms and concerns | Felt | Patient reports of symptoms or concerns and if these were resolved by care provision | Problems and Needs in Palliative Care - short version (PNPC-sv) | No time frame stated for unmet need measurement | Physical; Psychological; Spiritual; Practical; Autonomy; Social; Financial/legal | |
| Effendy et al. (2015) | No | 3) Sufficiency of service provision to resolve symptoms and concerns | Felt | Patient reports of symptoms or concerns and if these were resolved by care provision | Problems and Needs in Palliative Care - short version (PNPC-sv) | No time frame stated for unmet need measurement | Physical; Psychological; Spiritual; Autonomy; Financial/legal | |
| Hasegawa et al. (2016) | Partially | 3) Sufficiency of service provision to resolve symptoms and concerns | Felt | Patient reports of symptoms or concerns and if these were resolved by care provision | Supportive Care Needs Survey - Short Form (SCNS-SF34) | Past month | Physical; Psychological; Information; Healthcare; Sexuality | |
| Hasegawa et al. (2021) | No | 2) Access to services | Felt | Family members' reports of service use | Unnamed questionnaire | No time frame stated for unmet need measurement | Physical; Practical | |
| Huang et al. (2020) | No | 3) Sufficiency of service provision to resolve symptoms and concerns | Felt | Patient reports of symptoms or concerns and if these were resolved by care provision | Supportive Care Needs Survey - Short Form (SCNS-SF34) | Past 2-6 months | Physical; Psychological; Information; Healthcare; Sexuality | |
| Husain et al. (2013) | No | 3) Sufficiency of service provision to resolve symptoms and concerns | Felt | Patient reports of symptoms or concerns and if these were resolved by care provision | Supportive Care Needs Survey - Short Form (SCNS-SF34) | Past month | Physical; Psychological; Information; Healthcare; Sexuality | |
| Hwang et al. (2004) | No | 3) Sufficiency of service provision to resolve symptoms and concerns | Felt | Patient reports of symptoms or concerns and if these were resolved by care provision | Unnamed questionnaire | Last month of life | Physical; Psychological; Financial/legal; Healthcare; Social | |
| Jeyasingam et al. (2008) | Partially | 3) Sufficiency of service provision to resolve symptoms and concerns | Felt | Patients and family members' reports of symptoms or concerns related to activities of daily living | Screening Tool Activities of Daily Living (ST-ADL) | No time frame stated for unmet need measurement | Physical | |
| Johnsen et al. (2013) | Partially | 3) Sufficiency of service provision to resolve symptoms and concerns | Felt | Patient reports of symptoms or concerns and if these were resolved by care provision | Three Levels of Needs Questionnaire (3LNQ) | Past week | Physical; Psychological; Practical; Social; Sexuality | |
| Kavalieratos et al. (2014) | No | 1) Symptoms and concerns  2) Access to services | Felt; Comparative | Patient reports of unresolved symptoms; care service use data | McCorkle Symptom Distress Scale; Medical records | No time frame stated for unmet need measurement | Physical; Psychological | |
| Khan et al. (2012) | No | 3) Sufficiency of service provision to resolve symptoms and concerns | Felt | Patient and family members' reports of symptoms or concerns and if these were resolved by care provision | Problems and Needs in Palliative Care - short version (PNPC-sv) | No time frame stated for unmet need measurement | Physical; Psychological; Spiritual; Information; Autonomy; Social; Financial/legal | |
| Miniotti et al. (2019) | No | 3) Sufficiency of service provision to resolve symptoms and concerns | Felt | Patient reports of symptoms or concerns, sufficiency of care and desire for further attention | Supportive Care Needs Survey - Short Form (SCNS-SF34) | Past month | Physical; Psychological; Practical; Information; Sexuality | |
| Munn et al. (2006) | No | 3) Sufficiency of service provision to resolve symptoms and concerns | Felt | Family members' and healthcare staff reports of personal care needs and if these were resolved by care provision | Unnamed questionnaire | No time frame stated for unmet need measurement | Physical | |
| Oh et al. (2019) | No | 3) Sufficiency of service provision to resolve symptoms and concerns | Felt | Patient reports of supportive care needs that were not resolved | Amyotrophic Lateral Sclerosis Supportive Care Needs Instrument (ALSSCN) | Past month | Physical; Psychological; Spiritual; Practical; Information; Social | |
| Osse et al. (2005) | Partially | 3) Sufficiency of service provision to resolve symptoms and concerns | Felt | Patient reports of symptoms or concerns and if these were resolved by care provision | Problems and Needs in Palliative Care (PNPC) | No time frame stated for unmet need measurement | Physical; Psychological; Spiritual; Role activities; Financial/legal; Social; Autonomy; Healthcare | |
| Park et al. (2017) | Partially | 3) Sufficiency of service provision to resolve symptoms and concerns | Felt | Patient reports of spiritual needs and whether these were met | Unnamed questionnaire | No time frame stated for unmet need measurement | Spiritual | |
| Pearce et al. (2012) | Partially | 3) Sufficiency of service provision to resolve symptoms and concerns | Felt | Patient reports of spiritual needs and whether these were met | Unnamed questionnaire | No time frame stated for unmet need measurement | Spiritual | |
| Rachakonda et al. (2015) | Yes ("*there is a discernible, often ignored under-evaluated care-management gap in palliative cancer care, where the estimated clinical outcome is seldom translated into a patient-centered benefit. These supportive care needs that call for immediate attention are classified as 'unmet needs'.*") | 3) Sufficiency of service provision to resolve symptoms and concerns | Felt | Patient reports of symptoms or concerns and their continued needs for care | Needs Assessment for Advanced Cancer Patients (NA-ACP) | No time frame stated for unmet need measurement | Physical; Psychological; Spiritual; Information; Financial/legal; Social | |
| Rhodes et al. (2012) | No | 3) Sufficiency of service provision to resolve symptoms and concerns | Felt | Family members' reports of how well care provided matched the decedent's and family's needs | Family Evaluation of Hospice Care (FEHC) survey | No time frame stated for unmet need measurement | Physical; Psychological; Spiritual | |
| Schenker et al. (2014) | No | 3) Sufficiency of service provision to resolve symptoms and concerns | Felt | Patient reports of symptoms or concerns and if these were resolved by care provision | Unnamed questionnaire | Past month | Physical; Psychological; Information; Spiritual; Social | |
| Strupp et al. (2018) | No | 1) Symptoms and concerns | Felt | Patient reports of symptoms or concerns and their care needs in relation to how severely affected they are by their disease | Unnamed questionnaire | No time frame stated for unmet need measurement | Open question about aspects in which the patient wishes for more help or support | |
| Szekendi et al. (2016) | Partially | 1) Symptoms and concerns; 2) Access to services | Normative | Routine data including diagnosis and care service use | Medical records | No time frame stated for unmet need measurement | Physical | |
| Teno et al. (2004) | No | 3) Sufficiency of service provision to resolve symptoms and concerns | Felt | Family members' reports of symptoms or concerns and if these were resolved by care provision | Structured interview | Final 3 days of life or less | Physical; Psychological | |
| Teno et al. (2007) | No | 3) Sufficiency of service provision to resolve symptoms and concerns | Felt | Family members' reports of symptoms or concerns and if these were resolved by care provision | Family Evaluation of Hospice Care (FEHC) survey | No time frame stated for unmet need measurement | Physical; Psychological | |
| Teno et al. (2011) | No | 3) Sufficiency of service provision to resolve symptoms and concerns | Felt | Family members' reports of symptoms or concerns and if these were resolved by care provision | Family Evaluation of Hospice Care (FEHC) survey | No time frame stated for unmet need measurement | Physical; Autonomy | |
| Trandel et al. (2019) | No | 3) Sufficiency of service provision to resolve symptoms and concerns | Felt | Patient reports of symptoms or concerns and if these were resolved by care provision | Supportive Care Needs Survey - Short Form (SCNS-SF34) | Past month | Physical; Psychological; Information; Healthcare; Sexuality | |
| Wang et al. (2021) | No | 3) Sufficiency of service provision to resolve symptoms and concerns | Felt | Patient and family members' reports of symptoms or concerns and if these were resolved by care provision | Problems and Needs in Palliative Care - short version (PNPC-sv) | No time frame stated for unmet need measurement | Physical; Psychological; Information; Spiritual; Practical; Autonomy; Social; Financial/legal | |
| Wang et al. (2023) | No | 1) Symptoms and concerns | Felt | Patient reports of symptoms or concerns | Palliative Outcome Scale (POS) | Past week | Physical; Psychological; Spiritual; Information; Practical | |
| Watson et al. (2019) | No | 3) Sufficiency of service provision to resolve symptoms and concerns | Felt | Patient reports of symptoms or concerns and if these were resolved by care provision | Modified version of Supportive Care Needs Survey - Short Form (SCNS-SF34) | Past month | Physical; Psychological; Information; Healthcare; Sexuality; Financial/legal | |
| Wegier et al. (2021) | Partially | 1) Symptoms and concerns | Felt | Patient and family members' reports of symptoms or concerns | Edmonton Symptom Assessment Scale Revised (ESAS-r); Sudore's Advance Care Planning (ACP) Engagement Survey | At the time of measurement | Physical; Psychological; Information | |
| **Systematic reviews** | | | | | | | |  |
| Bore et al. (2024) | Yes ("*Unmet needs for supportive care services represent the gap between a cancer patient’s desire or need for specific services and their received experiences*.") | Not applicable due to no direct measurement of unmet palliative care needs | Felt |  |  |  |  | |
| Chen et al. (2020) | No |  | Felt |  |  |  |  | |
| Fu et al. (2020) | Yes ("*Unmet supportive care needs reflect the disparity between the supports that an individual perceives as necessary and those that are actually provided*.") |  | Felt |  |  |  |  | |
| Harrison et al. (2009) | Yes ("*Needs that were not addressed and where additional support was required were classified as ‘unmet needs’. … Unmet needs assessment adds a further dimension to needs assessment by distinguishing how well needs have been met and identifying those that remain unmet.*") |  | Felt |  |  |  |  | |
| Hart et al. (2022) | No |  | Felt |  |  |  |  | |
| Moghaddam et al. (2016) | Yes ("*In the context of supportive care, unmet needs reflect incongruity between the supports that an individual perceives to be necessary versus the actual supports provided*.") |  | Felt |  |  |  |  | |
| Schmidt et al. (2023) | Yes ("*The definition of unmet healthcare needs was constructed stepwise, first defining healthcare needs as what patients and the population as a whole desire to receive from healthcare services to improve overall health. Unmet needs were defined as needs that are either not addressed or receive insufficient attention.*") |  | Felt |  |  |  |  | |
| Ventura et al. (2014) | Yes ("*Needs go unmet when basic requirements to maintain quality of life have not been met. For patients, unmet needs tend to exist across practical, emotional, physical and existential domains.*") |  | Felt |  |  |  |  | |
| Wang et al. (2018) | Yes ("*Unmet needs assessment is designed to identify how well and how much their needs have been satisfied or not.*") |  | Felt |  |  |  |  | |

**Notes**: ^1^ Study also reported in Halkett et al. 2015; ^2^ Study also reported in Kirkland et al. 2022 & Kruhlak et al., 2021; ^3^ Study also reported in Buzgova et al., 2015

* To increase readability, we do not denote studies using the third approach as also using the first approach, even though both include measurement of symptoms and concerns. In the third approach, symptom measurement is part of the process of measuring unmet palliative care needs and not indicative of unmet needs in itself (as it is in approach 1).

‡ Domains based on Goni-Fuste et al.’s (24) framework of comprehensive needs assessment in palliative care: *Physical* (key indicators include pain, breathlessness, function/daily living activities), *Psychological* (Depression, anxiety, isolation), *Spiritual* (meaning of life, acceptance of dying, religious support), *Social* (support from social network, maintaining relations, express feelings with others), *Information* (information about diagnosis, prognosis and care options), *Financial/legal* (financial concerns, handling financial and legal arrangements, e.g., wills), *Practical* (ability to maintain personal hygiene, household tasks), *Autonomy* (dependency on others, experiencing loss of control, privacy), *Role activities* (difficulties with employment or studies, difficulty caring for children), Personal issues (handling personal affairs), and *Healthcare* (support from healthcare professionals, side effects, quality of care), as well as the added domain ‘Sexuality’ (sexual dysfunction, loss of libido).
